# Thermal Adaptation of the Archaeal and Bacterial Lipid Membranes

**DOI:** 10.1155/2012/789652

**Published:** 2012-08-15

**Authors:** Yosuke Koga

**Affiliations:** Department of Chemistry, University of Occupational and Environmental Health, Yahata-nishi-Ku, Kitakyushu 807-8555, Japan

## Abstract

The physiological characteristics that distinguish archaeal and bacterial lipids, as well as those that define thermophilic lipids, are discussed from three points of view that (1) the role of the chemical stability of lipids in the heat tolerance of thermophilic organisms: (2) the relevance of the increase in the proportion of certain lipids as the growth temperature increases: (3) the lipid bilayer membrane properties that enable membranes to function at high temperatures. It is concluded that no single, chemically stable lipid by itself was responsible for the adaptation of surviving at high temperatures. Lipid membranes that function effectively require the two properties of a high permeability barrier and a liquid crystalline state. Archaeal membranes realize these two properties throughout the whole biological temperature range by means of their isoprenoid chains. Bacterial membranes meet these requirements only at or just above the phase-transition temperature, and therefore their fatty acid composition must be elaborately regulated. A recent hypothesis sketched a scenario of the evolution of lipids in which the “lipid divide” emerged concomitantly with the differentiation of archaea and bacteria. The two modes of thermal adaptation were established concurrently with the “lipid divide.”

## 1. Introduction 

The unique structural characteristics of the archaeal polar lipids, that is, the *sn*-glycerol-1-phosphate (G-1-P) backbone, ether linkages, and isoprenoid hydrocarbon chains, are in striking contrast to the bacterial characteristics of the *sn*-glycerol-3-phosphate (G-3-P) backbone, ester linkages, and fatty acid chains. This contrast in membrane lipid structures between archaea and bacteria has been termed the “lipid divide” [1]. Because this has been repeatedly discussed [2–4], it is not discussed again here. The only thing that needs to be pointed out is that the enantiomeric difference of the lipid backbone (G-1-P and G-3-P), which is the most important feature from the evolutionary point of view, is insignificant in terms of the thermal adaptation of the membrane, because enantiomers have equivalent thermal properties.

The chemical properties and physiological roles of archaeal lipids are often discussed in terms of the presence of the chemically stable ether bonds in thermophilic archaea. However, based on the archaeal lipids analyzed thus far, as shown by lipid component parts analysis [5], the mesophilic archaea possess essentially the same core lipid composition as that of the thermophilic archaea. The ether bonds therefore do not seem to be directly related to thermophily. 

What are the most crucial distinguishing physiological characteristics of the archaeal and bacterial lipids? What are the distinctive features of thermophilic lipids? These questions will be discussed in the present paper from three distinct perspectives:

1. the relationship of the chemical stability of lipids with the heat tolerance of thermophilic organisms,
2. the increase in proportion of certain lipids as the growth temperature rises,
3. the lipid bilayer membrane properties that enable membranes to function at high temperatures.

Recently a hypothesis was published [1] on the differentiation of archaea and bacteria and the establishment of the “lipid divide.” The present paper will discuss two fundamental aspects of the thermal adaptation of microorganisms in relation to the domain differentiation and the emergence of the “lipid divide.” 

A preliminary form of the present discussion was first presented as a part of our previous review paper [6]. Driessen and Albers [7] have presented similar conclusion about membrane adaptations to high temperatures in relation to the membrane mechanisms of energy metabolism. The main conclusion of these two articles almost identical, even though the papers were independently prepared and the findings discussed from originally different point of view. 

## 2. The Chemical Stability of Lipids and the Heat Tolerance of Thermophilic Organisms

Because the ether bonds of archaeal lipids are for the most part not broken down under conditions in which ester linkages are completely methanolyzed (5% HCl/MeOH, 100°C for 3 hr), it is generally believed that the archaeal ether lipids are thermotolerant or heat resistant. This implies that thermophilic organisms are able to grow at high temperature due to the chemical stability of their membrane lipids. 

**Figure 1:**
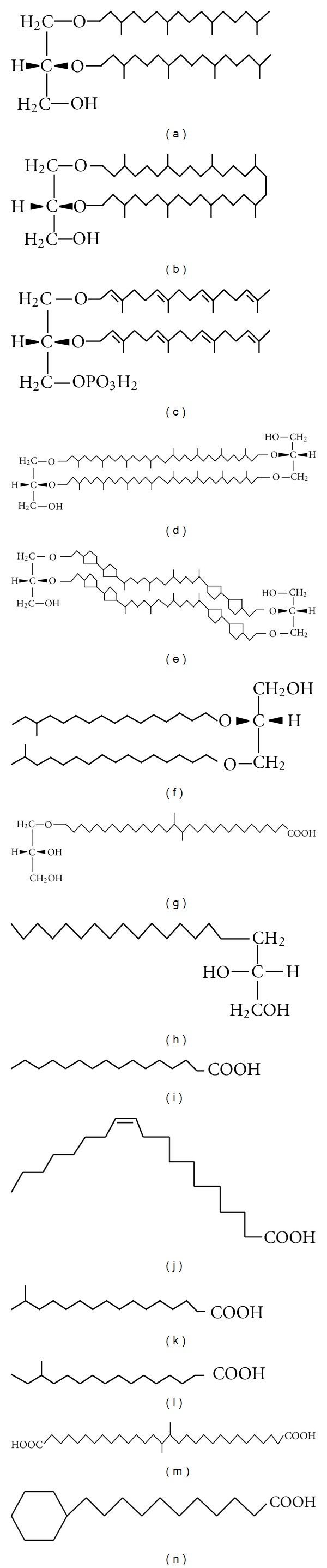
Structures of thermophilic lipid candidates (a) diphytanylglycerol (archaeol: archaeal diether lipid); (b) cyclic archaeol; (c) digeranylgeranylglycerophosphate (DGGGP); (d) caldarchaeol (archaeal tetraether lipid); (e) cyclopentane-containing caldarchaeol; (f) bacterial dither lipids; (g) 15,16-dimethyl-30-glyceryloxytriacontanoic acid; (h) 1,2-di-hydroxynonadecane (long-chain diol lipid); (i) palmitic acid (saturated straight chain fatty acid); (j) *cis*-vaccenic acid (monounsaturated straight chain fatty acid); (k) iso-C17 fatty acid; (l) anteiso-C17 fatty acid; (m) 15,16-dimethyltriacontandioic acid (diabolic acid); (n) 11-cyclohexylundecanoic acid. (a)–(e) Archaeal lipids; (f)–(n) bacterial lipids.

Figure 1 illustrates structures of some so-called “thermophilic” lipid candidates referred to in the following text.

Ether lipids (Figure 1(a)) are always present in the archaea that reside in high-temperature environments without exceptions, but the mesophilic archaea also have ether lipids. In fact, not only archaea but also certain thermophilic bacteria contain ether lipids. The thermophilic lipid candidates, in addition to the archaeal ether lipids, are the chemically stable monobranched fatty alcohol-containing diether lipids (Figure 1(f), *Thermodesulfobacterium commune* (optimum growth temperature: 70°C [8]) and *Aquifex pyrophilus* (85°C [9]); long chain dicarboxylic fatty acids (diabolic acid, Figure 1(m)) and 15,16-dimethyl 30-glyceryloxytriacontanoic acid (Figure 1(g)) from *Thermotoga maritima* (90°C [10]) and *Fervidobacterium islandicum* (75°C [11]); a long chain 1, 2-diol (Figure 1(h), (*Long chain diol lipid: in this lipid it can be considered that the first three carbons, C1 to C3, play the role of the backbone (instead of glycerol) of the lipid. The OH at the C1 may bind a polar head group and the OH at the C2 binds the first hydrocarbon chain, and the C3 and C4 represent a C–C-bond between the “backbone” and the remainder part of the long chain.)) from *Thermomicrobium roseum* (75°C [12]); cyclohexyl fatty acid (Figure 1(n)) from *Bacillus acidocaldarius* (65°C [13]). These have been assumed to be thermophilic lipids because of their thermostability (unhydrolyzability) (diether or C–C bond in the long-chain diol or membrane-spanning nature (dicarboxylic acid) like tetraether lipids). As a matter of fact, all the thermophilic archaea possess ether lipids, but not all of the organisms possessing the so-called “thermophilic” lipids shown above are themselves thermophilic. The same structure of diabolic acid was also found in *Butyrivibrio* sp. (39°C [14]); and cyclohexyl fatty acid in *Curtobacterium pusillum* (27°C [15]). Many species of the mesophilic methanoarchaea [5] have ether core lipids. On the other hand, some of the thermophilic organisms are able to survive with ester lipids in their membranes [10–13].

Because tetraether type, membrane-spanning polar lipids (Figures 1(d) or 1(e)) were first found in thermoacidophilic archaeon [16], these lipids are considered thermophilic lipids. Tetraether lipids are extended as a result of their C40 hydrocarbon chains passing across the membrane bilayer. Thus, tetraether lipids link the leaflets of the lipid bilayer covalently and thus make the membrane rigid. This structure allows membranes to tolerate extreme conditions. However, some of the nonthermophilic methanoarchaea have the same tetraether lipids [5]. *Methanothermobacter thermautotrophicus* (65°C) has both archaeol- and caldarchaeol-based lipids, while the mesophilic species of *Methanobacterium* (37°C) has almost the same core lipid composition. Similarly, some archaea that have caldarchaeol-based (tetraether-type) polar lipids in addition to archaeol-based polar lipids grow above 85°C, and there is one that grows at 20°C. Some archaea have only archaeol-based (diether-type) polar lipids and grow below 40°C, yet there is one that grows at 90°C. The hyperthermophilic *Methanopyrus kandleri* (90°C [5]) has also only diether-type polar lipids. The distribution pattern of the archaeol- and caldarchaeol-based polar lipids make it clear that these ether lipids are not absolutely required for tolerance of high temperature.

Archaeal ether lipids are synthesized from G-1-P and geranylgeranylpyrophosphate (GGPP). The first and second ether-bonded intermediates in the archaeal phospholipid synthesis pathway are geranylgeranylglycerophosphate (GGGP) and digeranylgeranylglycerophosphate (DGGGP, Figure 1(c)) [17, 18], respectively, which are allyl ether compounds. The allyl ether compounds are just as labile as or even more labile than the ester compounds; they are broken down *in vitro* at 5% HCl/MeOH, 80°C for 1 hr. Ether bonds themselves are stable, but their biosynthetic precursors are as labile as ester compounds. Since organisms with ester lipids are heat sensitive, Archaea cannot grow well at a high temperature, because the heat-sensitive biosynthetic intermediates, which are only present in a small amount, are easily broken down, so ether lipids cannot be synthesized.

Even if the chemically stable lipids that are present in thermophiles are indeed thermophilic lipids, it has not yet been made clear how such chemical stability specifically affects the response to high temperatures by thermophilic organisms. It is not yet apparent whether the ether lipids were specifically adapted for the purpose of survival at high temperatures.

## 3. Increases in the Proportion of Certain Lipids as the Growth Temperature Rises

In considering thermophilic lipids, not only chemical but also biological aspects are essential to an understanding of their activity. Lipids that increase in proportion to an increase in growth temperature may thus aptly be designated “thermophilic lipids.”

The fatty acid composition of a bacterium changes depending on the growth temperature. In *Escherichia coli*, unsaturated fatty acids (Figure 1(j)) increase along with a downshift in the growth temperature [19]. In *Bacillus* spp. and other bacterial species, isofatty acids (Figure 1(k)) increase along with an increase in the growth temperature, and anteiso fatty acids (Figure 1(l)) increase along with a lowering of the growth temperature [20–23]. The increasing fatty acids are often not a single fatty acid but rather a group of different fatty acids. A mesophilic strain of *Bacillus megaterium* [24] has been shown to have 25% iso-C15 and nearly 50% anteiso-C15 fatty acids at 20°C and 35% iso-C15 and 15% anteiso-C15 at 60°C. By contrast, a thermophilic strain of the organism can only grow between 45 and 70°C and the iso-C15 content (30–50%) is always higher than that of anteiso-C15 (lower than 10%). Furthermore, the growth of a psychrophilic strain of the bacterium is restricted to temperatures between 5 and 45°C, and the content of anteiso-C15 (around 50%) is always higher than that of iso-C15 (10–30%). The thermophilic or psychrophilic strains do not appear to regulate the branched chain C15 fatty acid content. This suggests that the iso-C15 fatty acid is thermophilic and the anteiso-C15 fatty acid is psychrophilic in this bacterial species.

In the extremely thermophilic methanoarchaea *Methanocaldococcus jannaschii*, when the growth temperature increases from 45°C to 65°C, the diether lipids (archaeol-based lipids) decrease from 80% to 20%, while the standard caldarchaeol-based and cyclic archaeol-based (Figure 1(b)) lipids increase from 10% to 40%, respectively [25].

The changes in the hydrocarbon composition of membrane lipids have a nature of lawfulness, but the mode of hydrocarbon composition change is different from species to species. Therefore, the pattern of fatty acid composition found in a given species, for example, *E*. *coli*, should be applied to other organisms only with the greatest caution. To find the actual underlying pattern in these phenomena, another point of view would seem to be required, and this is discussed in the next section.

## 4. Lipids as Cell Membrane Constituents Having a Permeability Barrier and Liquid Crystalline State

The third conceptualization of thermophilic/heat-tolerant lipids is based on a rather different point of view. Because lipids do not function as single molecules but as a membrane, that is, as an enormous number of molecules acting together, which assemble into a biologically functioning organelle, thermophilic lipids should be understood as lipids that normally function as a membrane at a high temperature. This is not achieved by chemical stability alone. At the moment that a lipid membrane came to enclose the cell contents, the real cell as we know it was born. With that event, cell membranes partitioned the inner cytoplasmic compartment away from their surroundings. From this time onward, membranes effectively functioned as a permeability barrier, controlling the in-flow and out-flow of low-molecular-weight compounds. This is the most primitive and essential function of a cell membrane. When cells became enclosed by such a membrane having this sort of permeability barrier, the cells achieved a distinct “individuality” and hence began to compete with the other individual cells in order to survive within the local community, and thus natural selection came more sharply into play. Therefore, the lipid constituents that enable the membrane to function as a highly permeable barrier at high temperatures are designated thermophilic lipids. 

Another essential general feature that is required for lipid membrane function is the capacity to persist in the liquid crystalline phase. The phase-transition temperature of the archaeal lipid membranes is far lower than that of fatty acyl ester lipids, reportedly being between –20 and –15°C [26]. The phase transition temperature of the normal fatty acyl ester phospholipid membrane is in a far higher temperature range (40–50°C) than the archaeal lipids, and this is dependent on their chain length, number of double bonds and the methyl branching position. Therefore, the archaeal polar lipid membrane can be presumed to be in liquid crystalline phase in the temperature range of 0 to 100°C, the range at which most archaea grow (biological temperature), while fatty acyl diester lipid membrane is in either a gel phase or liquid crystalline phase in the same temperature range, depending on their fatty acid composition.

In some archaea, the hydrocarbon chain properties are regulated by the number of cyclopentane rings (Figure 1(e), *Sulfolobus solfataricus*) [27] or the ratio of caldarchaeol/cyclic archaeol/archaeol (*Methanocaldococcus jannaschii*) [25]. The content of the transunsaturation of the isoprenoid chains was reported to decrease with a higher growth temperature in *Methanococcoides burtonii* [28]. However, the organism *Methanopyrus kandleri* has a sufficient number of double bonds in the isoprenoid chains in spite of its much higher growth temperature. Unsaturation is not related in a straightforward manner with the adaptation to low temperatures, which occurred in archaea. 

 One characteristic property of the archaeal lipid membrane is the extremely low permeability of solutes [29–32]. In addition, the permeability increases only slightly as the temperature goes up in the 0 to 100°C range. 

In contrast to the tetraether lipid liposomes, the fatty acyl ester lipid liposomes exhibit a low permeability at a low temperature, but the permeability drastically increases as the temperature increases [29]. The experimental results suggest that highly branched isoprenoid chains are a major cause of the low permeability of liposomes, but this phenomenon does not depend on the ether or ester bonds between the glycerophosphate backbone and hydrocarbon chains.

Bacteria grow at a temperature just above the phase-transition temperature at which membrane lipids are in a liquid crystalline state and retain a minimal level of permeability. The permeability of fatty acyl ester lipid membranes is highly temperature dependent and their phase-transition temperature is dependent on the fatty acid composition, so when the growth temperature shifts, the fatty acid composition of membrane lipids is quickly regulated. The phenomena described in Section 3 (regulation of the composition of unsaturated/saturated fatty acids (Figures 1(i) and 1(j)) in *E*. *coli*, and iso/anteiso fatty acids in *Bacillus* spp.) are explained by this mechanism. On the other hand, the isoprenoid ether lipids in the archaeal membrane are in a low permeability liquid crystalline state throughout the possible growth temperature range (0–100°C) [33], and even if the growth temperature changes, the two requirements are met without any need of a biological regulation mechanism.

Because isoprenoid ether lipid membranes are in the liquid crystalline phase and have a low permeability at biological temperatures, archaea are found living at temperatures as low as 1°C and as high as 100°C with the same archaeol and caldarchaeol lipid composition in the membrane. This is the most fundamental characteristic of the archaeal lipid membranes. Bacterial membranes can be characterized by the highly developed regulatory mechanisms they employ to meet the two conditions. We can see actual examples in the case of the hyperthermophilic *Pyrococcus furiosus* (optimum temperature, 98°C) [34], moderately thermophilic *Methanothermobacter thermautotrophicus* (65°C) [35], mesophilic *Methanobacterium formicicum* (37°C) [5] and *Methanogenium cariaci* (23°C) [5]. They all have nearly the same core lipid composition. Unsaturated archaeol (geranylgeranyl group-containing archaeol) is present in the psychrophilic *Methanococcoides burtonii* that can grow at 2°C [28] as well as the hyperthermophilic *Methanopyrus kandleri* (98°C) [36]. A lipid that can be utilized at both high and low temperatures because of its liquid crystalline phase and low permeability at a wide range of temperatures is aptly termed a “heat tolerant” lipid. 

On the other hand, bacterial fatty acyl ester lipid membranes should only function at the lowest temperature at which both a liquid crystalline state and low permeability are retained. This condition may be met at a temperature close to and above its phase transition temperature. Therefore, many bacteria with ester lipids control their fatty acid composition so as to meet these conditions. The control mechanism varies from species to species. In *Escherichia coli*, unsaturated fatty acids are maximal at lower growth temperatures. However, unsaturation is not the only mechanism to adapt to lower temperatures. In *Bacillus* spp., temperature adaptation is regulated by changing the iso/anteiso fattyacid composition [22].

 The archaeal lipid membrane does not have to regulate its hydrocarbon composition to meet the two conditions for temperature adaptation, because the two conditions are already in place at such a wide range of temperatures. 

## 5. Evolutionary Significance of Two Modes of Thermal Adaptation

Recently, a hypothesis [1] was put forward that provides an account for the differentiation of Archaea and Bacteria from the last universal common ancestor (LUCA) by means of the enantiomeric phase separation of the glycerophosphate backbones of the membrane lipids facilitated by their different hydrocarbon chains and diastereomeric structures. In LUCA cells, at least four different kinds of core lipids (Ai, Bf, Af, and Bi) are made up of a combination of G-1-P (A) or G-3-P (B) as the phospholipid backbone and isoprenoid (i) or fatty acid (f) as the hydrocarbon chains. The archaeal Ai membranes and bacterial Bf membranes thrive differently depending on the nature of their constituent lipids. Soon after the beginning of the differentiation of LUCA, the phase separation of various core lipids was still incomplete in the membrane. As the differentiating membranes become further purified, either mode of thermal adaptation may be established. This might act as a positive selection pressure. The two different modes of thermal adaptation evolved in parallel with the emergency of the “lipid divide” and Archaea-Bacteria differentiation. The “lipid divide” was produced not only by the physicochemical phase separation of the membrane lipids but also by the different forms of thermal adaptation. Accordingly, these two different organisms have adapted to a variety of environments.

## 6. Conclusion

It is concluded that no single, chemically stable lipid by itself was sufficient for the adaptation to living at high temperatures. Therefore, an alternative account of the emergence of heat-tolerant lipids has been presented. This view emphasizes the functioning of organismal lipids as membranes. Vitally functioning cell membranes must have at least two characteristics: a high permeability barrier and the capacity to maintain the liquid crystalline phase. Archaeal membranes are able to meet these conditions by means of isoprenoid chains, which are functional over the entire biological temperature range and do not require a regulatory mechanism to adapt lipids to changes in the environmental temperature. Because bacterial membranes have a temperature-dependent permeability and fatty acid composition-dependent phase transition over the complete biological temperature range, they have elaborate mechanisms by which they regulate the fatty acid composition at temperatures just above the phase transition temperature. It is not unusual perhaps should not be surprising that no change in the lipid composition of archaea took place as the growth temperature changed. The presence of the transdouble bonds in isoprenoid chains does not directly entail the adaptation of organisms to low-temperature environments. Instead, the two modes of thermal adaptation are the result of the early evolution of membrane lipids that enabled the differentiation of archaea and Bacteria by means of the establishment of the “lipid divide”.
